# The development and psychometric properties of oral health assessment instruments used by non-dental professionals for nursing home residents: a systematic review

**DOI:** 10.1186/s12877-020-01989-8

**Published:** 2021-01-09

**Authors:** Rojina Thapa, Ritesh Chimoriya, Amit Arora

**Affiliations:** 1 School of Health Sciences, Western Sydney University, Campbelltown Campus, Locked Bag 1797, Penrith, NSW 2751 Australia; 2 School of Medicine, Western Sydney University, Campbelltown Campus, Locked Bag 1797, Penrith, NSW 2751 Australia; 3 Translational Health Research Institute, Western Sydney University, Locked Bag 1797, Penrith, NSW 2751 Australia; 4 Oral Health Services, Sydney Local Health District and Sydney Dental Hospital, NSW Health, Surry Hills, NSW 2010 Australia; 5 Discipline of Child and Adolescent Health, Sydney Medical School, Faculty of Medicine and Health, The University of Sydney, Westmead, NSW 2145 Australia

**Keywords:** Oral health, Geriatric assessment, Psychometrics, Reliability, Validity, Non-dental professionals

## Abstract

**Background:**

Globally, oral health status of the geriatric population residing in nursing homes is poor. The integration of non-dental professionals is vital to monitor oral health, early identification and triaging of oral health problems, and timely referral to dental professionals. The aims of this systematic review were to provide a summary on the development and characteristics of oral health assessment instruments currently used by non-dental professionals for nursing home residents, and to perform a critical appraisal of their psychometric properties.

**Methods:**

This review was conducted as per the PRISMA guidelines. CINHAL (EBSCO), Medline (Ovid), and EMBASE (Ovid) were searched systematically. Two reviewers independently screened the title, abstract, and full text of the studies as per the eligibility criteria. Studies describing oral health assessment instruments used to assess oral health of nursing home residents by non-dental professionals were included. Using a methodological framework, each instrument was evaluated for purpose, content, and psychometric properties related to validity, reliability, feasibility, generalisability, and responsiveness. Additionally, the reporting quality assessment of each included study was performed according to the SURGE guidelines.

**Results:**

Out of the 819 screened articles, 10 studies were included in this review. The 10 identified instruments integrated 2 to 12 categories to assess oral health, which was scored on a 2 to 5-point scale. However, the measurement content varied widely, and none were able to comprehensively measure all aspects of oral health. Three measurement approaches were identified: performance- based assessment, direct inspection of the oral health status, and interview measures. Only eight instruments provided quality assessment on the basis of validity, reliability, feasibility and generalisability, whereas three instruments- Brief Oral Health Status Examination, Dental Hygiene Registration, and Oral Health Assessment Tool reported good methodological quality on at least one assessment criteria.

**Conclusions:**

None of the instruments identified in this review provided a comprehensive assessment of oral health, while three instruments appeared to be valid and reliable. Nonetheless, continuous development of instruments is essential to embrace the complete spectrum of oral health and address the psychometric gaps.

**Supplementary Information:**

The online version contains supplementary material available at 10.1186/s12877-020-01989-8.

## Background

There is a two-way relationship between oral health and general health [1]. Oral health refers to the condition of individual’s teeth and gums, and the health of the muscles and bones present in the mouth [2]. Impaired oral health may lead to pain, discomfort, reduced chewing ability, limited food choices, poor nutritional intake, low self-esteem, social avoidance, and has a negative impact on the quality of life [3]. Moreover, poor oral health outcomes such as dental caries (tooth decay) and periodontal (gum) diseases may further increase the risk of systemic diseases [4–6].

Especially in the geriatric population, age-related degenerative changes, risk of chronic diseases, physical weakness, functional dependency, cognitive impairment, and behavioural problems act as contributing factors in the development of oral health problems [7]. Furthermore, polypharmacy, lack of dexterity, multiple systemic conditions such as diabetes, dementia, and obesity put the older adults at a high risk of oral diseases [8]. As a result, numerous cases of dental caries, xerostomia (dry mouth), oral mucosal lesions, periodontal diseases, oro-dental trauma, oral cancers, and frequent tooth loss are seen in older adults [9]. Several studies conducted globally suggest that about 1 in 5 older adults aged 65 and above have tooth loss [10–12], while more than half have periodontal diseases [11, 13]. This suggests that oral health problems are one of the major global health concerns with an increased prevalence of oral diseases among older people [14].

Globally, oral health status of older adults residing in nursing homes is poor [15–17]. This is because regular oral health examinations may not be available in Residential Aged Care Facilities (RACFs) and frequent commuting may be difficult due to limited mobility, cognitive impairment, and communication issues [18, 19]. It is estimated that up to 80% of nursing home residents do not receive daily oral care as they depend on the care staff as a result of their cognitive and physical limitations [20]. Studies have shown that more than 40% of the nursing home residents in Norway had unsatisfactory oral hygiene [21]; and about 70% did not receive adequate oral care in Sweden [22]. Similarly, a study conducted in Hong Kong revealed that the mean number of decayed, missing, and filled teeth (DMFT) in institutionalised older adults was 21.4, while it was only 17.7 for those non-institutionalised [23]. This suggests that among older people, those who are residing in nursing homes are at a particularly higher risk of developing oral health problems.

Oral health assessment of all residents on a regular basis is a promising approach to delivering high quality oral health services in RACFs [24–26]. There has been a growing emphasis on providing oral health training particularly for non-dental professionals such as nursing and care staff working in RACFs [25, 27, 28]. Moreover, it is essential to ensure that oral health promotion programs are tailored to the needs of older adults and are focused on capacity building of non-dental professionals so that knowledge is effectively translated into practice [23, 29]. The integration of front-line health care providers is also vital to monitor resident’s oral health, early identification and triaging of oral health problems, and timely referral to dental professionals [30].

As most oral health assessment instruments had been developed specifically for use by dental professionals, they may be too complex for use by non-dental professionals [31]. Few instruments have been developed for use by non-dental professionals such as Oral Assessment Guide (OAG) [32] and the Holistic and Reliable Oral Assessment Tool (THROAT) [33]; however, their primary focus is on hospital and rehabilitation settings and have not been tested in residential care settings. Some of the oral health assessment instruments designed for use in RACFs include Brief Oral Health Status Examination (BOHSE) [34], Activities of Daily Oral Hygiene (ADOH) [35], Mucosal Plaque Score (MPS) [36], and Oral Health Assessment Tool (OHAT) [37]. In order to provide credible evidence to inform clinical practice and oral health policies, assessing and testing the psychometric properties of these instruments is essential. These include: validity- the extent to which an instrument measures what it is intended to measure [38], reliability- the extent to which the measurement is consistent and free from errors [39], feasibility- the administration of the instrument and the requirements associated with it [40], generalisability- the application of the instrument in different populations and settings [40], and responsiveness- the ability of an instrument to identify the important clinical changes over time within the individual [41].

In 2005, a systematic review of oral health assessment by nurses and carers for residents with dementia in RACFs was conducted [24]. The study indicated a shortage of validated and reliable tools available for use by non-dental professionals in RACFs. However, several oral health assessment instruments have been developed in the last two decades, and although a few of these instruments have been tested on their reliability and validity [33, 42, 43], a summary of the instruments’ development and psychometric properties have not been published. Therefore, the aims of this systematic review were to provide a summary on the development and characteristics of oral health assessment instruments currently used by non-dental professionals for nursing home residents, and to perform a critical appraisal of the psychometric properties related to validity, reliability, feasibility, generalisability, and responsiveness of these instruments. Additionally, this review also assessed the reporting quality of the existing literature addressing the development and validation of these instruments.

## Methods

This review was conducted in accordance with the Preferred Reporting Items for Systematic Reviews and Meta-Analysis (PRISMA) guidelines [44] (Additional file 1: Appendix 1). The protocol of this systematic review was registered with PROSPERO International Prospective Register of Systematic Reviews (2020: PROSPERO CRD42020134034) [45].

### Eligibility criteria

#### Inclusion criteria

- Written in English language.
- Studies targeting the geriatric population of RACFs.
- Studies describing tools used by non-dental professionals.
- Studies focused on tools used to assess oral health.

#### Exclusion criteria

- Studies focused on the geriatric population in community settings.
- Studies indicating tools used solely by oral health professionals such as dentists and dental hygienists.
- Studies describing tools used solely in hospitals and rehabilitation units.
- Studies focused solely on the oral health related quality of life.
- Studies focused exclusively on the population groups with specific medical conditions.

### Information sources

A large-scale search was conducted in three electronic databases- CINHAL (EBSCO), Medline (Ovid), and EMBASE (Ovid) using the specified search strategy without any restrictions on publication date (i.e. from the time of inception to present) and study type. Further, reference lists of all articles identified from the electronic databases were screened and a manual search was performed for previous systematic reviews. The initial search was conducted from 5 March 2019 and then updated on 12 August 2020. The studies were restricted to English language publications.

### Search strategy

The Population Intervention/Exposure Comparator Outcome Study design (PICOS) [46] criteria were applied to design the key concepts and related additional terms. A combination of specific medical subject headings (MeSH), terms and keywords related to oral health, the geriatric population, and RACFs were devised with the assistance from an expert Health Sciences Librarian (Additional file 2: Appendix 2). The Boolean operators ‘and’ and ‘or’ were used to narrow down and widen the search scope. The pilot search was pre-tested in the Medline (Ovid) database and was subsequently adapted to the syntax and subject headings of the other databases employed. The search strategy is provided in Additional file 3: Appendix 3.

### Study selection

All studies retrieved from the electronic databases were exported to a reference manager software Endnote X9 for elimination of duplicates, screening, and selection. Two reviewers (RT and AA), in accordance with the eligibility criteria, independently screened the searches and filtered the manuscripts by title and abstract relevance. Studies that intended to measure the oral health status of the geriatric population residing in RACFs or to develop a new oral health assessment tool used by non-dental personnel were read in full text. Any disagreements were resolved through discussion with a third reviewer (RC). The studies that were read in full text and found not to meet the inclusion criteria have their reason/s for exclusion reported in Additional file 4: Appendix 4.

### Data extraction process

A standardised data extraction form was developed to evaluate all oral health assessment instruments, using a methodological framework [47, 48] established for the evaluation of health assessment indices as a reference. Necessary adaptations were made to the categories within the framework so that the appraisal was relevant for oral health assessment instruments. The data extraction form was pilot tested on two studies to ensure it met the review objectives and all relevant information were recorded consistently. Data from all included studies were extracted independently by two reviewers (RT and AA). Information on country of origin, publication year, authors, type of tool, purposes, developers, method of development, administration procedure, estimated duration for assessment, and scoring categories for each assessment tool were extracted. Furthermore, studies were assessed for psychometric analysis, and information related to validity, reliability, feasibility, generalisability, and responsiveness were extracted.

### Assessment of reporting quality

The reporting quality assessment of each included study was performed according to the specifications of the Reporting Guidelines for Survey Research (SURGE) [49]. The SURGE is an adequate and appropriate tool to appraise the reporting quality in surveys and to gain detailed information on the characteristics of the survey instruments used [50]. The studies were appraised in terms of eight categories: background, methods, sample selection, characteristics of the research tool, results, response rates, interpretation and discussion, and ethics and disclosure [49]. The degree to which the intention of each study matched were then reported as partial, inadequate, and adequate. Two reviewers (RT and AA) independently assessed the methodological quality.

### Data synthesis

Following data extraction, a narrative was created to provide a descriptive synthesis of the included studies in two steps. The first task was to assess the purpose and content of all identified instruments including sampling frame, settings, and oral health domains targeted in each study. The second task was to ascertain the psychometric properties- reliability, validity, feasibility, generalisability, and responsiveness of each instrument.

#### Validity

The determination of validity for a particular instrument depends upon a variety of contexts and the purpose of the research [40]. It is important to define content, face, and construct validity for a newly developed instrument [39]. Content validity is associated with the process of planning and developing an instrument and looks at the extent to which the content of the instrument reflects the concept that is being measured [40]. Face validity is where an instrument appears to test what it is intended to measure. Construct validity assesses the degree to which an instrument measures what it is supposed to measure and can be assessed through confirmatory factor analysis, hypothesis testing, and comparing and examining instrument associations with existing instruments [40].

#### Reliability

Various ways of estimating reliability include assessing internal consistency of each item in the instrument (internal consistency reliability), consistency of measurement over time (test-retest reliability), establishing degree of agreement between either two or more examiners (inter-rater reliability), and assessing result consistency measured by the same examiner (intra-rater reliability) [39].

#### Feasibility

Feasibility can be judged based on parameters such as estimated time, resources required, and to what extent the instruments are suitable to the users and recipients [51].

## Results

### Results of the search

**Fig. 1:**
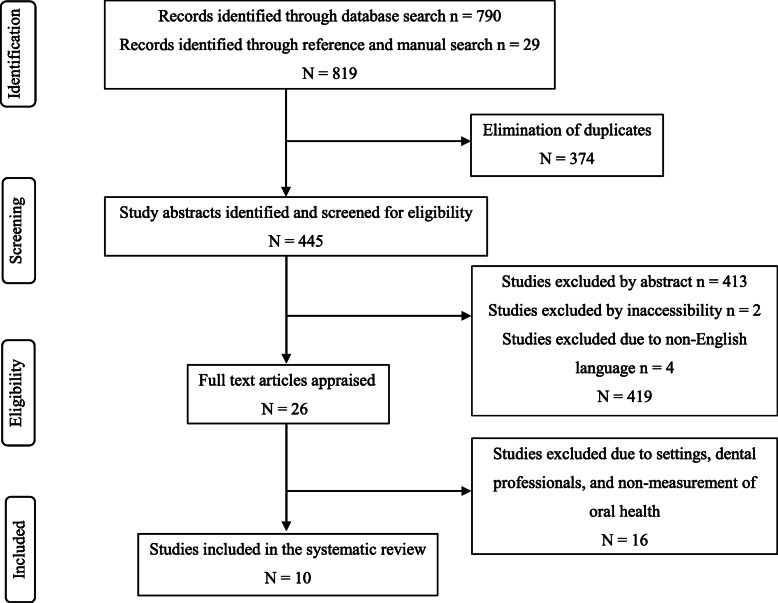
PRISMA flow diagram of literature search and study selection process

A total of 819 articles were retrieved from the electronic databases and manual search. Of these, 374 articles were eliminated due to duplication. After reviewing the abstracts, 413 articles were excluded as they did not measure oral health and only reported on the development and validation of oral health instruments used in RACFs. Further 4 studies were removed due to publication only in non-English language, and 2 studies were removed due to accessibility issues, despite repeated attempts to contact the authors. A total of 26 full-text studies were assessed by two reviewers (RT and AA), which resulted in further exclusion of 16 studies based on the eligibility criteria. The reasons for exclusion are presented in Appendix 4. Finally, a total of 10 studies were included in this systematic review. The results of the search process are outlined in the PRISMA flow diagram (Fig. 1).

### Characteristics of oral health assessment instruments

Table 1 illustrates the overview of oral health assessment instruments included in this review. The 10 identified instruments were developed between 1990 and 2017. All included studies originated from high income countries such as Sweden [56], Norway [36, 52], United States [34, 35, 53, 55], Australia [43], and Japan [31, 54]. All instruments integrated 2 to 12 categories to assess oral health, which was scored on a 2 to 5-point scale.

**Table 1:** Overview of oral health assessment instruments

Instrument	Year	Country of origin	Authors	Type of instrument
ADOH [35]	2001	United States	Bauer et al.	4 categories scored on 5-point scale
BOHSE [34]	1995	United States	Kayser-Jones et al.	10 categories scored on 3-point scale
DHR [52]	2016	Norway	Fjeld et al.	2 categories scored on 3-point scale
GOHAI [53]	1990	United States	Atchison et al.	12 categories scored on 3-point scale
MPS [36]	1999	Norway	Henriksen et al.	2 categories scored on 4-point scale
OAS [54]	2017	Japan	Shizuko et al.	9 categories scored on 3-point scale
OHAT [43]	2004	Australia	Chalmers et al.	8 categories scored on 3-point scale
OHSTNP [31]	2017	Japan	Tsukada et al.	12 categories scored on 3-point scale
RAI-MDS [55]	1990	United States	Morris et al.	2 sections with yes/no questions
ROAG-J [56]	2016	Sweden	Johansson et al.	8 categories scored on 4-point scale

ADOH: Activities of Daily Oral Hygiene, BOHSE: Brief Oral Health Status Examination, DHR: Dental Hygiene Registration, GOHAI: Geriatric Oral Health Assessment Index, MPS: Mucosal Plaque Score, OAS: Oral Assessment Sheet, OHAT: Oral Health Assessment Tool, OHSTNP: Oral Health Screening Tool for Nursing Personnel, RAI-MDS: Resident Assessment Instrument-Minimum Data Set, ROAG-J: Revised Oral Assessment Guide-Jonkoping

The characteristics of oral health assessment instruments are outlined in Table 2. The primary purpose of all included studies was to develop an instrument for nurses to assess oral health of aged care residents. Additionally, six studies [34, 36, 43, 52–54] stated testing and validation of instruments by nurses and care workers in RACFs as their primary objective. Most of the instruments were developed by a panel of experts in geriatrics, dentistry, nursing, and in consultation with the users. Four instruments were developed by modifying the existing instruments- OHAT from BOHSE [43]; Dental Hygiene Registration (DHR) from Simplified Oral Hygiene Index (OHI-S), MPS, and Revised Oral Assessment Guide (ROAG) [52]; Revised Oral Assessment Guide-Jonkoping (ROAG-J) from ROAG and OAG [56]; and Oral Health Screening Tool for Nursing Personnel (OHSTNP) from OHAT and Oral Screening Sheet [31]. In terms of administration, most instruments used inspection and palpation for examination of oral health status and scored using a point scale. Geriatric Oral Health Assessment Index (GOHAI) [53] used questionnaire-based oral health assessment, while ADOH [35] used task-performance oriented questions to assess oral health.

**Table 2:** Characteristics of oral health assessment instruments

Tools	Purpose	Expertise of developers	Development	Administration	Scoring
**ADOH** [35]	To assess the physical ability to manipulate the aids used in oral self-care and to measure the return to function in response to care intervention and rehabilitative services.	Panel of expertise from geriatric dentistry.	Conceptualised from the classification scheme used in medicine i.e. Activities of Daily Living.	The sequence of interviewing instructions is given in the assessment instrument to guide the examiner in monitoring the individual’s abilities in performing each task.	4 categories scored on 5-point scale from 0–4. 0: for performing each step without any help; 1: requires a device to enhance the performance; 2: expends 50% or more effort in task completion; 3: expends less than 50% effort in task completion; 4: for total assistance in performing task. Total score of 16. Classifies an individual as independent, partly dependent and dependent.
**BOHSE** [34]	To evaluate the oral health status of both cognitively impaired and unimpaired residents by nursing staff.	Registered nurses, dentists, certified nursing assistants, and licensed vocational nurses.	Developed through a review of available oral assessment guides, consultation with dental faculty, and recommendations from American Dental Association.	Examination is carried out in the same order as given in instrument guide starting from inspection and palpation of lymph nodes to observing oral cleanliness. Tongue blades, light, disposable gloves, and gauze squares were used as per need.	10 categories scored on 3-point scale from 0 to 2. 0: indicating healthy end; 2: unhealthy end of the scale. Total score of 20 which ranges from 0: very healthy to 20: very unhealthy. A higher cumulative score reflects the presence of many oral health problems.
**DHR** [52]	To develop easy and quick dental hygiene assessment scale for institutional nurses.	Panel of dentists, dental hygienists, nurses, and geriatricians.	Literature review on existing instruments. Benefits and strengths from OHI-S, MPS, and ROAG were considered and DHR criteria were discussed with a panel of expertise.	Assessment is conducted using a pen torch light source. Entire tooth surfaces of upper jaw are examined first and then the lower jaw for the presence of plaque (yes/ no).	2 categories scored on 3-point scale from 0 to 2. Total score of 4, where 0: teeth without plaque represent optimal; 1: visible plaque on one or more teeth represent increased risk; 2–4: visible plaque on more than half of the teeth represent risk of oral health diseases. Lower and upper jaw are scored separately.
**GOHAI** [53]	To gather information for easy diagnosis and provide appropriate interventions by patient self-report measure.	Expertise from geriatric dentistry, community dentistry, public health dentistry, and nursing departments.	Developed on the basis of past oral health status measurements, review of literature on impacts of oral diseases, existing questionnaire on oral functional status and symptoms, and consultation with health care providers.	As it is a self-reported assessment, geriatric oral health assessment is conducted through an interview.	12 categories scored on 3-point scale from 1 to 3. 1: always, often; 2: sometimes, seldom; 3: never. High score represents good oral health and low score represents oral health problems.
**MPS** [36]	To evaluate the oral health and oral hygiene in hospitals and other institutions.	Group of experts from gerontology and dentistry faculty.	Not stated.	Examination is performed in normal daylight with the aid of an artificial light source. Intraoral examination is performed with the help of dental mirrors.	2 categories scored on 4- point scale from 1 to 4. Total score of 8, where 2–4: acceptable; 5–6: unacceptable; 7–8: poor. Mucosal and Plaque Score are calculated separately.
**OAS** [54]	To enhance the oral health of older adults who need nursing care.	Expertise comprising of dentists, dental hygienists, medical social workers, and medical doctors.	Oral assessment items were decided by the focus group of expertise after the literature review on assessment of oral health.	Oral examination is performed without using any special instrument.	9 categories scored on 3-point scale from A-C A: poor oral condition which needs immediate improvement; B: an optimal level, yet improvement is needed; C: condition with minimum problems. Grade A, B and C represent Score 2, 1 and 0. High score indicates poor oral health, which requires professional interventions.
**OHAT** [43]	To assess the oral health status by carers in residential care facilities.	Group of expertise from geriatric dentistry, dementia care, nurses, dentists, dental hygienists, carers, and RACFs.	Developed by modifying BOHSE after review of literature on oral assessment tools and suggestions from the group of expertise from Australia and United States.	Not stated.	8 categories scored on 3-point scale from 0 to 2. 0: healthy; 1: oral changes; and 2: unhealthy. Total score is calculated by summing scores from each category.
**OHSTNP** [31]	To assist nursing staff of long-term care in identifying dental referral needs.	Group of nurses, dentists, and caregivers.	Developed by combining and modifying OHAT and Oral Screening Sheet.	General inspection by using a penlight, tongue depressor, and dental mirrors.	12 categories scored on 3-point scale from 0 to 2. 0: good; 1: fair; and 2: poor. Questions related to the need for referral and reason of assessment are added at the bottom of the screening tool.
**RAI-MDS** [55]	To collect minimum amount of data regarding resident’s strengths, needs, and potential risk to plan and monitor individualised care in long term care setting.	Clinicians and researchers from nursing, social work, medicine, physical, occupational and speech therapy, and nutrition disciplines.	Developed by extensive review and revision of the assessment instrument and developing multiple drafts of MDS consulting with experts and a basic testing of the instrument.	Nurses complete the assessment form through resident’s records, direct observation, and conversation with residents.	2 sections pertaining to oral health consisting of yes/no items. All responses indicating potential oral problems requires automatic dental referral.
**ROAG-J** [56]	To assess the oral health status of elderly people in a daily nursing care.	Not stated.	Developed by revising ROAG, which in turn is revised from OAG.	Examination is performed following the manual provided in ROAG-J.	8 categories scored on 4-point scale from 0 to 3. 0 and 1: no intervention; 2: preventive care action by nurses; 3: requires dentist for treatment.

ADOH: Activities of Daily Oral Hygiene, BOHSE: Brief Oral Health Status Examination, DHR: Dental Hygiene Registration, GOHAI: Geriatric Oral Health Assessment Index, MPS: Mucosal Plaque Score, OAS: Oral Assessment Sheet, OHAT: Oral Health Assessment Tool, OHSTNP: Oral Health Screening Tool for Nursing Personnel, RAI-MDS: Resident Assessment Instrument-Minimum Data Set, ROAG-J: Revised Oral Assessment Guide-Jonkoping

The following section summarises the development and characteristics of each oral health assessment instrument included in this review.

#### Activities of Daily Oral Hygiene (ADOH)

ADOH was developed to determine and monitor the functional dependency of an individual to operate the aids used in oral self-care [35]. The four domains for assessing the dependency function of oral self-care activities include flossing, tooth brushing, topical fluoride application, and oral rinses. A score ranging from 0 to 4 is allocated for each task, which takes about 5 to 15 min to complete. For edentulous older adults, brushing and oral rinsing tasks are rated with the total dependency score of 8. On the basis of the total score, an individual is classified as independent, partly dependent, or fully dependent.

#### Brief Oral Health Status Examination (BOHSE)

BOHSE was developed to measure the oral health conditions of cognitively impaired and unimpaired residents of aged care facilities by the care providers [34]. The ten assessment items related to oral health and function include lymph nodes, lips, tongue, tissue inside cheek, floor and roof of the mouth, gums between teeth and/or under the dentures, saliva, condition of natural teeth and dentures, pairs of teeth in chewing position, and oral cleanliness. Each item consists of three descriptors and is scored from 0 to 2. The summative score ranges from 0 (very healthy) to 20 (very unhealthy).

#### Dental Hygiene Registration (DHR)

DHR is a dental hygiene assessment scale designed to describe the individual’s dental hygiene and to evaluate the nurse’s own performance in delivering dental hygiene [52]. DHR was developed considering the benefits and strengths of OHI-S, MPS, and ROAG. The presence of visible dental plaque on all tooth surfaces is examined and scored to assess oral hygiene. The upper and lower jaw are scored separately, and then summed to get an overall score of 0–4. If one of the jaws is edentulous, the other jaw is scored and multiplied by two.

#### Geriatric Oral Health Assessment Index (GOHAI)

GOHAI is a questionnaire-based tool designed to assess the oral health of older adults [53]. It consists of twelve items related to physical function, psychological function, and pain or discomfort; and includes both positive and negative items rated on a 3-point Likert scale as 1-always, often; 2- sometimes, seldom; and 3- never. It is recommended as a quick and easy instrument for use by nurses in RACFs as it helps to collect oral complaints and decide appropriate dentist referral [57]. GOHAI has been translated into different languages- Chinese [58], Dutch [59], French [60], Swedish [61], and Turkish [57] since its development.

#### Mucosal Plaque Score (MPS)

MPS measures only two parameters- degree of inflammation of the mucosa and the amount of plaque deposited around the teeth or on dentures, which facilitates a quick overall evaluation of the oral health condition and oral hygiene [36]. The mucosal and plaque score are evaluated separately on the basis of four criteria- normal appearance, mild inflammation, moderate inflammation, and severe inflammation; and no visible plaque, small amounts of hardly visible plaque, moderate amounts of plaque, and abundant amounts of plaque, respectively. The scores range from 1 to 4 for each parameter which gives a total MPS score ranging from 2 to 8.

#### Oral Assessment Sheet (OAS)

OAS measures the oral hygiene in terms of tooth and gingival cleanliness, coating of tongue, and bad breath; whereas oral function is measured in terms of jaw opening, tongue thrust, dry mouth, and chewing and biting function of teeth and dentures [54]. The examiner rates the oral hygiene, biting and chewing, and oral function each with three items using a grading scale: A- poor oral condition which needs immediate improvement; B- optimal oral condition yet some improvement is required; and C- oral condition with minimal problems. A numerical score 2, 1 and 0 is assigned to grade A, B and C respectively.

#### Oral Health Assessment Tool (OHAT)

OHAT is a modified version of BOHSE and evaluates the oral health status of residents of RACFs including those with cognitive impairment [43]. It was designed to measure the oral health status, monitor oral hygiene, and identify the referral need based on the eight specified categories- lips, tongue, gums and tissues, saliva, natural teeth, dentures, oral cleanliness, and dental pain. Each category is rated on a 3-point scale: 0- healthy, 1- oral changes, and 2- unhealthy. OHAT is an easy to use tool for non-dental personnel ranging from carers to nurses.

#### Oral Health Screening Tool for Nursing Personnel (OHSTNP)

OHSTNP was developed to identify the dental referral needs of residents in a long-term care facility [31]. It measures the oral health status and oral function and consists of twelve categories (A-L): A to G are related to oral health and are modified from OHAT; H to L are related to oral function and are modified from Oral Screening Sheet; and K and L indicate the response from residents or staff. Each category consists of three descriptors and are scored on a 3-point scale: 0 (good), 1 (fair), and 2 (poor).

#### Resident Assessment Instrument-Minimum Data Set (RAI-MDS)

MDS is an assessment instrument consisting of minimum number of items; whereas, RAI refers to the assessment system and guidelines of care planning [62]. RAI-MDS was revised to RAI-MDS version 2.0 by retaining the previously tested instrument while modifying and adding new items. It consists of seventeen sections (A-Q) with 400 data items related to health and care at an individual resident level. Among them, two sections of MDS are related to oral health section: L (oral/nutritional) and M (oral/dental). Oral/nutritional items include oral problems, height and weight, nutritional problems, and nutritional approaches; and oral/dental status include oral conditions in terms of debris, denture, natural teeth, inflammation of gums, cleanliness, and dental caries. The oral health items were not changed for MDS v2.0, except that they are now included in section K and L [63]. The responses that suggest oral problems or risk are marked as ‘•15’, which indicates dental referral according to the resident assessment protocol and a system of follow-up instructions [63]. It is currently used in Canada, United States, England, Germany, Japan, Australia, New Zealand, Sweden, Norway, France, Spain, and Belgium [64, 65].

#### Revised Oral Assessment Guide – Jonkoping (ROAG-J)

ROAG-J was developed to measure the oral health status of nursing home residents in daily nursing care. It is an updated version of ROAG [42], which is in turn revised from OAG [32]. ROAG-J describes the oral health by assessing the state of lips, voice, mucous membranes, gums, teeth, dentures, saliva, and tongue. It is rated on a 0–3 grade scale, where Grade 0 and 1 means no intervention is required, Grade 2 means an intervention is required by the nursing staff, and Grade 3 means an intervention is required by a dentist. The scores from all categories are summed up to get the potential score ranging from 0 to 27 and a higher score implies poor oral health [56].

### Psychometric properties of oral health assessment instruments

Table 3 summarises the psychometric analysis of oral health assessment tools. Out of the ten identified instruments, eight instruments- BOHSE [34], DHR [52], GOHAI [53], MPS [36], OAS [54], OHAT [43], OHSTNP [31], and RAI-MDS [55] provided quality assessment on the basis of validity, reliability, feasibility and generalisability. Overall, three instruments- BOHSE [34], DHR [52], and OHAT [43] reported good methodological quality on at least one assessment criteria and appeared to be valid and reliable assessment tools for use by non-dental professionals to assess the oral health of nursing home residents. The following section summarises the psychometric properties of each oral health assessment instrument included in this review.

**Table 3:** Psychometric analysis of oral health assessment instruments

Name of the instrument	ADOH [35]	BOHSE [34]	DHR [52]	GOHAI [53]	MPS [36]	OAS [54]	OHAT [43]	OHSTNP [31]	RAI-MDS [55]	ROAG-J [56]
**1. Content validity**										
a. Were the methods of selecting items appropriate?	Not stated	Yes	Not stated	Not stated	Not stated	Not stated	Yes	Not stated	Not stated	Not stated
b. Is the definition of what is being measured clearly specified?	No	Yes	Partially	No	Not stated	Not stated	Partially	Partially	Partially	Not stated
c. Were intended categories (i.e. relevant areas to be included and excluded) clearly stated?	Not stated	Partially	Partially	Not stated	Not stated	Not stated	Partially	Not stated	Not stated	No
d. Are all relevant components of each category included?	Not stated	Yes	Not stated	Not stated	Not stated	Not stated	Yes	Not stated	Not stated	Not stated
**2. Face Validity**										
a. On the face of it does it describe the intended purpose?	Not stated	Yes	Not stated	Not stated	Not stated	Not stated	Yes	Not stated	Not stated	Not stated
**3. Construct Validity**										
a. Does the instrument perform in expected ways when compared with other oral health assessment indices?	Not stated	Not stated	Yes, good correlation with OHI-S. Spearman’s correlation coefficient Rs = 0.78, and MPS Rs = 0.83.	Significant correlation with Single-item self-rating of dental health Pearson’s correlation coefficients r = 0.47	Not stated	Not stated	Not stated	Not stated	Not stated	Not stated
**4. Reliability**										
a. Has the reliability been measured?	No	Yes, Test-retest coefficients 0.79–0.88	Yes	Yes	Yes	Yes	Yes	Yes	Yes	No
b. Inter-rater reliability	No	< 0.80	1st time kappa (k) = 0.5 2nd time k = 0.8	No	Test 1 Weighted kappa (k) = 0.70; Test 2 k = 0.77	Not stated	Correlation coefficient = 0.74	Spearman’s correlation coefficient 0.81, 0.84 and 0.83	r = 0.46	No
c. Intra-rater reliability	No	No	1st time k = 0.7 2nd time k = 0.8	No	Weighted kappa k = 0.62	Not stated	Correlation coefficient = 0.78	No	Not stated	No
d. Internal consistency	No	No	No	Yes, Cronbach’s alpha α = 0.79	No	Yes, Cronbach’s alpha α = 0.72	No	No	Not stated	No
**5. Feasibility**										
a. Are special skills tools and/or training required?	No	In-service education	No	No	No	Yes	Yes	Yes	No	No
b. Is it easy to perform and administer?	Yes	Yes	Yes	Yes	Yes	Yes	Yes	Yes	Yes	Yes
c. How long does it take to perform?	5–15 min	Average 7–9 min	≤1 min	30 min	2–4 min	Not stated	Average 7.8 min	Average 114–202 s	Average 30 min	3–4 min

ADOH: Activities of Daily Oral Hygiene, BOHSE: Brief Oral Health Status Examination, DHR: Dental Hygiene Registration, GOHAI: Geriatric Oral Health Assessment Index, MPS: Mucosal Plaque Score, OAS: Oral Assessment Sheet, OHAT: Oral Health Assessment Tool, OHSTNP: Oral Health Screening Tool for Nursing Personnel, RAI-MDS: Resident Assessment Instrument-Minimum Data Set, ROAG-J: Revised Oral Assessment Guide-Jonkoping

#### Validity

Six instruments- ADOH [35], ROAG-J [56] OAS [54], RAI-MDS [55], OHSTNP [31] and MPS [36] did not establish validity of the instrument which showed inadequate quality in methodology. Only three instruments- BOHSE [34], GOHAI [53], and OHAT [43] reported on content validity; two instruments- BOHSE [34] and OHAT [43] reported on face validity; and one instrument- DHR [52] established construct validity.

DHR showed a good correlation with reported gold standards MPS [36] and Debris Index from OHI-S [52]. GOHAI showed a significant association with self-reported dental health; however, it showed a weaker correlation with clinical measures except for the number of teeth [53]. All items in OHAT [43] were not assessed and it did not reflect the construct to be measured comprehensively. In RAI-MDS 2.0, oral/dental items lacked validity as it under detects the oral/dental problems as compared to the clinical assessment by dental professionals [66].

#### Reliability

Two instruments- ADOH [35] and ROAG-J [56] did not report on reliability. Two instruments- OHAT [43] and BOHSE [34] assessed stability of the instrument by test-retest reliability. OHAT [43] failed to show correlations over time, assessments were repeated at 3 and 6 months, hence the methodological quality was poor. Whereas BOHSE [34] reported moderate sample size and unweighted kappas with high test-retest reliability. The percent agreement for the individual items of BOHSE varied from 50.5 to 98.0, and unweighted kappas ranged from 0.09 to 0.82, which showed a statistical significance [34]. Assessment of measurement properties on individual items of OHAT ranged from intra-rater reliability of 74.4% for oral cleanliness to 96.6% for a referral to the dentist; and unweighted kappas ranged from 0.51 to 0.80 indicating substantial agreement, whereas percent agreement between nurses ranged from 72.6% for oral cleanliness to 92.6% for dental referral and unweighted kappas varied from 0.48 to 0.80 showing substantial inter-rater agreement.

For DHR [52], inter-rater and intra-rater reliability were scored, where unweighted kappas was 0.7 for the dental hygienist and 0.8 for clinical nurse, which showed significant agreement between the examiners. However, oral/dental items in RAI-MDS [67] showed poor inter-rater agreement. The percent agreements between the examiners in OHSTNP were statistically significant for the categories E-L but the kappa values (0.05–0.20) and observed agreement (24.6–39.1%) for categories A-D were not significant [31]. Furthermore, two instruments- OAS (α = 0.72) [54], and GOHAI (α = 0.79) [53] indicated excellent internal consistency, which was assessed by calculating Cronbach’s alpha.

#### Feasibility

All instruments assessed the oral health status of the residents and required an examiner to administer all or some of the items in the assessment tools. Some tools required in-service education and training to perform the test, specifically for BOHSE [34], OHAT [43], OHSTNP [31], and OAS [54]. The estimated time required to complete the assessments ranged from a minimum of 1 min for DHR [52] to a maximum of 30 min for GOHAI [53].

#### Generalisability and responsiveness

GOHAI can be used by both dental professionals and non-dental personnel for the assessment of older adults [57, 68]. GOHAI is an internationally recognised tool, used in China, France, Sweden, United States, Netherlands, and Turkey [57, 59]. RAI-MDS has been implemented in the United States and Canada and can be used in different health care settings such as rehabilitative units, acute care, home care, and palliative care [62]. OHAT and BOHSE are widely accepted, validated, and user-friendly tools to be administered by non-dental personnel in American and Australian aged care facilities [24, 34]. Responsiveness of the oral health assessment instrument was measured in only one instrument- oral/dental items of RAI-MDS. There was no significant change in resident’s oral/dental problems over 6 years from 2007 to 2012 [66].

### Reporting quality of identified instrument studies

Among the 10 identified studies describing the oral health assessment instruments, the summary of reporting guidelines for survey research is compiled in Table 4. All studies adequately described the study objectives, results, and interpretation and discussion of the findings. Methodological concerns such as data entry, replication, and questionnaire administration were not described in all identified studies. Similarly, only one study discussed the role of response rate, non-response rate, and its calculation [53]. Furthermore, all studies provided information on the development of research tool except one [36], description of research tool except one [56], scoring methods except one [55], and reliability and validity except two [35, 56]; whereas, five studies [31, 34, 36, 53, 55] reported the instrument pre-testing features.

**Table 4:** Survey reporting quality of identified studies describing oral health assessment instruments

Reporting items	ADOH [35]	BOHSE [34]	DHR [52]	GOHAI [53]	MPS [36]	OAS [54]	OHAT [43]	OHSTNP [31]	RAI-MDS [55]	ROAG-J [56]
**Background**										
Literature review	P	A	A	A	P	A	A	P	A	A
Explicit research question	I	A	A	A	I	I	A	A	A	I
Clear study objectives	A	A	A	A	A	A	A	A	A	A
**Methods**										
Methods of data analysis	I	A	A	A	P	P	A	A	A	A
Questionnaire administration	I	I	I	P	I	I	I	I	I	I
Location of data collection	I	A	A	A	I	I	A	A	A	A
Dates of data collection	I	I	A	I	I	A	I	I	I	A
Methods sufficiently described for replication	I	I	I	I	I	I	I	I	I	I
Methods of data entry	I	I	I	I	I	I	I	I	I	I
**Sample selection**										
Sample size calculation	I	I	I	I	I	I	I	I	A	I
Method of sample selection	I	P	I	P	I	I	P	A	P	A
Description of population and sample frame	I	A	I	A	I	I	A	A	A	A
**Research Tool**										
Description of research tool	A	A	A	A	A	A	A	A	A	I
Development of research tool	P	A	A	A	I	A	A	A	A	A
Instrument pretesting	I	A	I	A	P	I	I	A	A	I
Instrument reliability and validity	I	A	A	A	P	A	A	A	P	I
Scoring methods	A	A	A	A	A	A	A	A	I	A
**Results**										
Results of research presented	P	A	A	A	P	A	A	A	A	A
Results address objectives	A	A	A	A	A	A	A	A	A	A
Generalisability	I	A	P	I	I	I	I	I	P	I
**Response rate**										
Response rate stated	I	I	I	A	I	I	I	I	I	I
Response rate calculated	I	I	I	A	I	I	I	I	I	I
Discussion of nonresponse	I	P	I	I	I	I	I	I	I	I
Missing data	I	I	I	I	I	I	I	I	I	I
**Interpretation and discussion**										
Interpret and discuss findings	A	A	A	A	A	A	A	A	A	A
Conclusions and recommendations	I	P	P	P	I	P	P	P	I	P
Limitations	I	P	A	I	I	I	I	I	I	I
**Ethics and discourse**										
Consent	I	A	A	I	I	A	A	A	I	A
Sponsorship	I	A	A	A	I	I	A	A	A	A
Research ethics approval	I	I	I	I	I	A	A	A	I	A

A: Adequate, P: Partial, I: Inadequate. ADOH: Activities of Daily Oral Hygiene, BOHSE: Brief Oral Health Status Examination, DHR: Dental Hygiene Registration, GOHAI: Geriatric Oral Health Assessment Index, MPS: Mucosal Plaque Score, OAS: Oral Assessment Sheet, OHAT: Oral Health Assessment Tool, OHSTNP: Oral Health Screening Tool for Nursing Personnel, RAI-MDS: Resident Assessment Instrument-Minimum Data Set, ROAG-J: Revised Oral Assessment Guide-Jonkoping

## Discussion

The objectives of this systematic review were to provide a summary on the development and characteristics of oral health assessment instruments currently used by non-dental professionals for nursing home residents, and to perform a critical appraisal of the psychometric properties related to validity, reliability, feasibility, generalisability, and responsiveness of these instruments. We found ten oral health assessment instruments that were developed and tested to assess oral health of nursing home residents. Most of the instruments were developed by a panel of experts in geriatrics, dentistry, nursing, and in consultation with the users. However, narrow content, poorly defined constructs for measurement, and psychometric weaknesses were identified in the oral health assessment instruments.

A wide variation in measurement content was found across the oral health assessment instruments. OAS [54] and OHSTNP [31] measure oral function; DHR [52] and MPS [36] measure dental plaque; ADOH [35] measure oral self-care function; OHAT [43], BOHSE [34], and ROAG-J [56] measure oral health status in terms of lips, gums, tongue, saliva, tissues, natural/artificial teeth and cleanliness; RAI-MDS [55] measures oral/dental and nutritional status; and GOHAI [53] measures oral health conditions and psycho-social and functional problems. However, oral health encompasses the condition of individual’s teeth and gums, and the health of the muscles and bones present in the mouth [2]. This indicates that none of the instruments were able to comprehensively measure all aspects of oral health.

Three major approaches for assessing oral health were identified: performance-based assessment, direct inspection of the oral health status, and interview measures. Performance-based assessment provides a quantitative measurement of an individual’s ability to perform oral health related activities. However, a limitation of this approach is that it may fail to differentiate incapable or uninterested individuals [68]. As tools such as ADOH focus on self-care activities, a high score does not necessarily indicate a good oral health, especially in individuals with poor oral hygiene and inadequate oral health literacy [35]. Direct inspection integrates the inspection of lips, gums, teeth, tissues, tongue, and mucous membranes for any signs of oral problems. However, an unbiased observation relies largely on the human judgement and assessment accuracy may be affected by the variability in examiner [69]. Interview measures is a cost-effective way to collect information on oral health problems of cognitively intact residents; however, it has limited applications in RACFs. Moreover, the chances of oral symptoms being omitted, under-rated, and exaggerated by the examiner in both direct inspection and interview approaches may create discrepancy leading to false conclusions [70].

The variation in cognitive level and behaviour problems among nursing home residents evolve complexity in oral health screening [71]. Moreover, challenges may arise due to the lack of communication, co-operation, and self-reporting [26]. Oral care can be improved in moderately impaired residents by targeting nurses and carers to enhance their oral health skills; whereas, stage-appropriate palliative oral care should be considered for severely impaired residents [72]. For unimpaired individuals with the ability to learn self-care skills, oral care function can be regained and maintained. Therefore, there is a need of an easy instrument to evaluate oral self-care of residents with cognitive impairment and such context should be considered through scientific approaches and research [73]. Instruments such as BOHSE [34], OHAT [43], and MPS [36] can be used for both cognitively impaired and unimpaired nursing home residents; GOHAI [53] is only applicable for cognitively unimpaired residents.

Methodological issues need to be reported transparently in the research process as it helps to assess the strengths and weaknesses and allow refinement of the instrument [50]. The reporting quality of the studies included in this review demonstrate mixed findings. Some domains such as background, results and its interpretation, and discussion were reported thoroughly; however, domains related to the methodological features were addressed inadequately. In most studies, the response rate and scoring issues of the instruments were not mentioned. Overall, all studies demonstrated inadequate compliance in reporting guidelines, which indicates possible advancement in developing oral health assessment instruments via further research.

Out of the ten identified instruments, only eight provided quality assessment on the basis of validity, reliability, feasibility and generalisability. However, the reporting of psychometric properties lacked explicit explanation on the concept of development of many instruments, which limits their methodological quality. Three out of the ten identified instruments- BOHSE [34], DHR [52], and OHAT [43] reported good methodological quality on at least one assessment criteria and appeared to be valid and reliable assessment tools for non-dental professionals to assess the oral health of nursing home residents. In a systematic review conducted in 2005, the authors found BOHSE as the most validated and reliable oral health assessment tool [24]. Although OHAT was developed as a modified form of BOHSE [43], oral health measurement categories of OHAT such as saliva, oral cleanliness, and dental pain require more investigation [43]. Similarly, OHSTNP [31], OAS [54], ADOH [35], RAI-MDS [55], and MPS [36] executed poor methodological quality of oral health measurement as the studies lacked comprehensive reflection of items to be measured (i.e. oral health). Furthermore, nurses were less proficient in accurately assessing lips, saliva, gums, mucosal membrane status, and chewing difficulty due to the lack of fundamental dental knowledge. Three studies [58, 60, 74] reported on the validation of GOHAI and three studies [53, 57, 61] described its reliability and validity. Nevertheless, further research is necessary to review some of the items and to determine the sensitivity of the instruments to changes due to interventions over time.

Non-dental professionals can perform oral health assessment and provide referral services for nursing home residents, when supported with adequate resources and training [75]. Although nursing staff and carers of RACFs recognise the importance of oral health assessment, they have raised concerns on time commitment [31]. Moreover, inadequate knowledge among non-dental professionals increases the risk of underestimation and overestimation of the symptoms leading to inappropriate scoring [70]. Therefore, some basic training for non-dental professionals on the specific signs and symptoms indicating oral infections and diseases is required [76]. Apart from training, enhancement of oral health knowledge of non-dental professionals is necessary. Provision of educational programs, use of diverse teaching formats, and regular reinforcement by a dental hygienist are found to be effective measures in enhancing the oral health of residents [77]. Moreover, some items of the oral health assessment instruments may require revision so that non-dental professionals can understand and administrate it easily, and further consideration is necessary in relation to frail and older adults in RACFs [78].

This systematic review has several strengths worth reporting. First, it provides valuable insight into the development, characteristics, and psychometric properties of oral health assessment instruments currently used by non-dental professionals for nursing home residents. Second, this review was conducted and reported in compliance with guidelines such as PRISMA and SURGE. Third, a structured methodological framework was used to retrieve data and critically appraise the existing oral assessment tools. Finally, the findings of our review may provide essential information for both dental and non-dental professionals, which may aid in the successful collaboration of both professionals to ensure better oral health outcomes for the geriatric population residing in RACFs.

There are a few limitations of this review. First, articles published in languages other than English were not included and it is therefore possible that we may have missed some instruments published in a non-English language. Second, we may have missed some relevant publications despite following the PRISMA guidelines and using a combination of specific MeSH, terms and keywords related to oral health, the geriatric population, and RACFs. Finally, two studies were removed due to accessibility issues despite repeated attempts to contact the authors, which could have provided valuable information. Nonetheless, this review presents an essential finding that none of the identified instruments were able to comprehensively measure all aspects of oral health. Further research is required on the development of valid and reliable instruments particularly for non-dental professionals that addresses the complexity, psychometric gaps, and appropriate content suitable for individuals utilising the tool.

## Conclusions

Older adults residing in nursing homes are at a particularly high risk of developing oral health problems. Research highlights the importance of non-dental professionals such as nursing and care staff in oral health assessment and promotion in residential settings. This systematic review succeeded in providing a summary on the development and characteristics of oral health assessment instruments currently used by non-dental professionals for nursing home residents and performing a critical appraisal of the psychometric properties of these instruments. The measurement content varied widely across the ten identified instruments, and none were able to comprehensively measure all aspects of oral health. Three measurement approaches were identified: performance- based assessment, direct inspection of the oral health status, and interview measures. Only eight instruments provided quality assessment on the basis of validity, reliability, feasibility and generalisability. However, three instruments- BOHSE, DHR, and OHAT appeared to be valid and reliable assessment tools for use by non-dental professionals to assess the oral health of nursing home residents. Nonetheless, continuous development of instruments is essential to address the psychometric gaps and embrace the complete spectrum of oral health. Moreover, there is a need of a valid, reliable, and more comprehensive tool to assess the oral health, identify the oral health needs, and generate positive outcomes in maintaining long-term oral health of the geriatric population residing in nursing homes.

## Supplementary Information

**Additional file 1: Appendix 1.** Prisma Checklist

**Additional file 2: Appendix 2.** PICOS (Search terms)

**Additional file 3: Appendix 3.** MEDLINE search strategy

**Additional file 4: Appendix 4.** Reasons for exclusion of studies

## Data Availability

The datasets generated during and/or analysed during the current study are available from the corresponding author on reasonable request.

## Abbreviations

ADOH: Activities of Daily Oral Hygiene
BOHSE: Brief Oral Health Status Examination
DHR: Dental Hygiene Registration
DMFT: Decayed-Missing-Filled-Teeth
GOHAI: Geriatric Oral Health Assessment Index
MPS: Mucosal Plaque Score
OAG: Oral Assessment Guide
OAS: Oral Assessment Sheet
OHAT: Oral Health Assessment Tool
OHI-S: Simplified Oral Hygiene Index
OHSTNP: Oral Health Screening Tool for Nursing Personnel
PRISMA: Preferred Reporting Items for Systematic Reviews and Meta-Analysis
PICOS: Population Intervention/Exposure Comparator Outcome Study Design
RAI-MDS: Resident Assessment Instrument-Minimum Data Set
ROAG: Revised Oral Assessment Guide
ROAG-J: Revised Oral Assessment Guide-Jonkoping
RACFs: Residential Aged Care Facilities
SLUMS: Saint Louis University Mental Status Examination
SURGE: Reporting Guidelines for Survey Research
THROAT: The Holistic and Reliable Oral Assessment Tool
