# Supplementary material for: The development and psychometric properties of oral health assessment instruments used by non-dental professionals for nursing home residents: a systematic review
Source: BMC Geriatr. 2021 Jan 9;21:35. doi: 10.1186/s12877-020-01989-8 (PMC7797120; doi:10.1186/s12877-020-01989-8)
Supplement: Supplementary file 2 — Additional file 2: Appendix 2. PICOS (Search terms) [file 12877_2020_1989_MOESM2_ESM.docx]

**Appendix 2.** PICOS (Search terms)

| Key Concepts | P (Population) | I (Intervention) | Co (Context) |
| --- | --- | --- | --- |
|  | Geriatric | Oral Health Assessment Tool | Residential Aged Care Facility |
| Additional terms | Aged  Elder*  Old*  Older adult  Dental care for aged  Geriatric dentistry | Dental Care  Dental hygiene  Oral health  Oral hygiene  Oro-facial pain  Oral health assessment  Assessment  Clinical assessment tools  Dental hygiene assessment  Geriatric assessment | Nursing home  Residential care facility  Residential care  Institutional care  Aged care facilities  Nursing facilities |
