# Supplementary material for: The development and psychometric properties of oral health assessment instruments used by non-dental professionals for nursing home residents: a systematic review
Source: BMC Geriatr. 2021 Jan 9;21:35. doi: 10.1186/s12877-020-01989-8 (PMC7797120; doi:10.1186/s12877-020-01989-8)
Supplement: Supplementary file 3 — Additional file 3: Appendix 3. MEDLINE search strategy [file 12877_2020_1989_MOESM3_ESM.docx]

**Appendix 3.** MEDLINE search strategy

| MeSH terms | | Results |
| --- | --- | --- |
| 1. | (MH “Aged) OR “aged” | 5,371,493 |
| 2. | “elder*” | 277,390 |
| 3. | “geriatric” | 110,787 |
| 4. | “old*” | 895,155 |
| 5. | 1 OR 2 OR 3 OR 4 | 5,889,925 |
| 6. | “dental health” | 17,942 |
| 7. | “dental” | 572,556 |
| 8. | (MH “Dental Care”) OR “dental care” | 44,963 |
| 9. | (MH “Oral Hygiene”) OR “oral hygiene” | 35,214 |
| 10. | (MH “Oral Health”) OR “oral health” | 43,647 |
| 11. | “oral care” | 21,358 |
| 12 | “oro-facial pain” | 116 |
| 13. | 6 OR 7 OR 8 OR 9 OR 10 OR 11 OR 12 | 585,891 |
| 14. | 5 AND 13 | 105,104 |
| 15. | (MH “Dental Care for Aged”) OR “dental care for aged” | 2,515 |
| 16. | “geriatric dentistry” | 1,685 |
| 17. | 14 OR 15 OR 16 | 105,552 |
| 18. | (MH “Clinical Assessment tools”) OR (MH “ Dental Hygiene Assessment”) OR “assessment” | 1,383,377 |
| 19. | “oral health assessment” | 529 |
| 20. | (MH “Geriatric Assessment”) OR “geriatric assessment” | 29,098 |
| 21. | 18 OR 19 OR 20 | 1,384,527 |
| 22. | (MH “Nursing Homes”) OR “nursing home” | 47,857 |
| 23. | (MH “Residential Facilities”) OR “residential care” | 8,279 |
| 24. | “institutional care” | 4,473 |
| 25. | “aged care facility” | 296 |
| 26. | “nursing facility” | 2,467 |
| 27. | (MH “Residential Care”) OR “residential care” | 8,341 |
| 28. | 22 OR 23 OR 24 OR 25 OR 26 OR 27 | 60,289 |
| 29. | 17 AND 21 AND 28 | 283 |
