# Supplementary material for: The development and psychometric properties of oral health assessment instruments used by non-dental professionals for nursing home residents: a systematic review
Source: BMC Geriatr. 2021 Jan 9;21:35. doi: 10.1186/s12877-020-01989-8 (PMC7797120; doi:10.1186/s12877-020-01989-8)
Supplement: Supplementary file 4 — Additional file 4: Appendix 4. Reasons for exclusion of studies [file 12877_2020_1989_MOESM4_ESM.docx]

| **Author/s and Year Published** | **Abbreviations** | | **Instruments** | **Reasons of exclusion** |  |
| --- | --- | --- | --- | --- | --- |
|  |  | |  |  |  |
| Andersson et al. (2002) | ROAG | | Revised Oral Assessment Guide | Focused on patient from geriatric rehabilitation ward. |  |
| Augsburger & Elahi (1982) | DPI | | Dental Plaque Index | Do not measure oral health status of older people. |  |
| Chamlers & Johnson (2004) | OHCP | | Oral Health Care Protocol | Focused on oral hygiene interventions. |  |
| Chen et al. (2017) | DAT | | Dental Activities Test | Focused in individuals with cognitive impairment. |  |
| Dickinson et al. (2001) | THROAT | | The Holistic and Reliable Oral Assessment | Used in hospitalized older people. |  |
| Delwel et al. (2018) | OPS-NVI | | Orofacial Pain Scale for Non-Verbal Individuals | Focused on people with dementia. |  |
| Eilers et al. (1988) | OAG | | Oral Assessment Guide | Focused on the Bone Marrow Transplantation (BMT) patient. |  |
| Greene & Vermillion (1960) | OHX | | Oral Health Index | Used by oral Health personnel in dental health practices and community settings. |  |
| Greene & Vermillion (1964) | OHI-S | | Simplified Oral Health Index | Used by oral health personnel. |  |
| Loe (1967) | GI | | Gingival Index | Used by oral health personnel |  |
| MacEntee et al. (1999) | CODE | | Clinical Oral Disorders in Elders | Used by dental professionals |  |
| Morris et al. (1997) | MDS-HC | | Minimum Data set for Home Care | Focused on older people living in community. |  |
| Paulsson et al. (2000) | OHEP | | Oral Health Education program | Focused on oral health care intervention. |  |
| Potting et al. (2006) | NNMSS | | Nijmegen Nursing Mucositis Scoring System | Used to identify the chemotherapy induced mucositis. |  |
| Slade & Spencer (1994) | OHIP | | Oral Health Impact Profile | Focused on quality of life |  |
| Van der Velden (2009) | DPSI | | Dutch Periodontal Screening Index | Used in general dental check-ups. |  |
|  | |  |  |  |  |
|  |  | |  |  |  |

**Appendix 4: Reasons for exclusion of studies**
